# Heterogeneous characters modeling of instant message services users’ online behavior

**DOI:** 10.1371/journal.pone.0195518

**Published:** 2018-05-07

**Authors:** Hongyan Cui, Ruibing Li, Yajun Fang, Berthold Horn, Roy E. Welsch

**Affiliations:** 1 State Key Lab of Networking and Switching Technology, Beijing University of Posts and Telecommunications, Beijing, P.R. China; 2 Beijing Lab of Advanced Information Networks, Beijing, P.R. China; 3 Key Lab of Network System Architecture and Convergence, Beijing, P.R. China; 4 Computer Science and Artificial Intelligence Laboratory, Massachusetts Institute of Technology, Cambridge, Massachusetts, United States of America; 5 Sloan School of Management, Massachusetts Institute of Technology, Cambridge, Massachusetts, United States of America; Hangzhou Normal University, CHINA

## Abstract

Research on temporal characteristics of human dynamics has attracted much attentions for its contribution to various areas such as communication, medical treatment, finance, etc. Existing studies show that the time intervals between two consecutive events present different non-Poisson characteristics, such as power-law, Pareto, bimodal distribution of power-law, exponential distribution, piecewise power-law, et al. With the occurrences of new services, new types of distributions may arise. In this paper, we study the distributions of the time intervals between two consecutive visits to QQ and WeChat service, the top two popular instant messaging services in China, and present a new finding that when the value of statistical unit *T* is set to 0.001s, the inter-event time distribution follows a piecewise distribution of exponential and power-law, indicating the heterogeneous character of IM services users’ online behavior in different time scales. We infer that the heterogeneous character is related to the communication mechanism of IM and the habits of users. Then we develop a combination model of exponential model and interest model to characterize the heterogeneity. Furthermore, we find that the exponent of the inter-event time distribution of the same service is different in two cities, which is correlated with the popularity of the services. Our research is useful for the application of information diffusion, prediction of economic development of cities, and so on.

## Introduction

The study of distribution characteristics of human behavior has a long history. For a long while, people have been using the Poisson distribution to quantify the model of human activities. A different opinion appeared in 2005, when Albert-László and Barabási published a paper titled as ‘the origin of bursts and heavy tails in human dynamics’ in Nature[1], which proposed that the distributions of time intervals between two consecutive events, called inter-event time, followed a heavy tailed distribution rather than the exponential distribution produced by the Poisson process. The new opinion is different from the traditional observation of human behavior and led to a study frenzy on human behavior. Most researchers thought the distributions of inter-event time fits a power-law distribution. For example, in paper [2] researchers use the freely available Wikipedia’s editing records and find that the time series of events whose inter-event times follows a probability distribution that displays a fat tail. Alexei Vázquez, et al. In paper [3] find the distribution of inter-event times (IETs) between two consecutive human activities exhibits a heavy-tailed decay behavior and the oscillating pattern with a one-day period, reflective of the circadian pattern of human life. Yadong Zhou, et al. found that the dynamic sizes of incidental topic groups followed a heavy-tailed distribution, and developed an adaptive parametric method for predicting the dynamics of incidental topic groups based on the finding in [4]. However, some researchers have different opinions. Malmgren R D, et al. mentioned that for the correspondence of sixteen famous writers, actors, politicians and scientists from the middle of sixteenth century to the middle of twentieth century, the inter-event time distribution can be better described by a cascading Poisson process than other kinds in [5]. Stouffer D B, et al. [6] thought that the lognormal distribution better describes e-mail communications. In [7], László Gyarmati and Tuan Anh Trinh showed that users’ time spent online fit Weibull distributions whereas the duration of user’s online session fit a power-law distribution. Chenxu Wang, et al. found that the distribution of inter-event times of microblog posting and wiki revising followed a piecewise distribution, and they proposed that the human dynamics were heterogeneous in different time scales in [8], [9]. There are obvious characteristics of circadian rhythm [10], burstyness [11], and memorability [12] in human behavior, which may explain the heavy-tailed distribution. Modeling is one of the best ways to reveal the pattern of human behavior. Based on the existing results, the models used to quantify human behavior can be roughly divided into three classes: 1) model based on queuing theory; 2) model based on memory [13], interest [14], rhythm [15] or some other ingredients; 3) model based on social interaction [16]. Besides direct analysis of temporal statistical characteristics of human behavior, there is some work aiming at the statistical characteristics over time of systems driven by human [17]–[19].

There may be a relationship between mobile Internet services usage behavior and other human behavior. For example, in [20] Yuanyuan Qiao, Xiaoxing Zhao, Jie Yang, and Jiajia Liu proposed that app usage had a strong relationship with human mobility. In [21] Fengli Xu, Yong Li, Min Chen, and Sheng Chen found a link between cyberspace and the physical world with social ecology. Furthermore, researchers in [22] proposed that the behavior pattern of IM services users is closely correlated with the development of the economy, transportation, and communication in the same area. For research on human dynamics, researchers focus on the inter-event time distribution of different activities. From existing studies, we can see that some different types of distributions are proposed to describe the inter-event time distribution. With the emergence of new services, human behavior may show some different temporal characteristics and thus need to be described with new types of distributions. In China, QQ and WeChat are two of the most popular mobile Internet services. Both of them belong to IM (Instant Messaging) services. People can send messages, have voice or video chat with friends, write logs, and so on by using QQ and WeChat. On average, billions of records can be produced by QQ and WeChat users in major cities every day. So the analysis of the temporal characteristics of QQ and WeChat users’ online behavior is useful for research on human dynamics. In this paper, we focus on analyzing the temporal characteristics of QQ and WeChat users’ online behavior in two developed cities Chongqing(City-A) and Tianjin(City-B) in China. The research can promote the study of human behavior and the prediction of a city’s economic condition.

The paper’s structure is as follows: Sect. II gives a brief introduction of our real data set and data processing. Sect. III analyzes the temporal characteristics of QQ users’ online behavior. Sect. IV analyzes the temporal characteristics of WeChat users’ online behavior. Sect. V analyzes the relationship between the inter-event time distribution and the popularity of IM services. In Sect. VI we propose a combination model to describe the heterogeneous characteristics of IM services users’ online behavior and verify the accuracy of the combination model using the Ali-talk data. In Sect. VII we summarize our conclusions from the paper.

## Data processing

Our raw datasets (A, B, C) were obtained from China Unicom. The dataset A consists of 171,734,242 records produced by all of QQ users and 30,070,724 records produced by all of WeChat users in Chongqing China from Nov 17th, 2012 to Nov 21st, 2012, six days in total. The dataset B covers 55,942,780 records produced by all of QQ users and 366,288,051 records produced by all of WeChat users in Tianjin China from May 4th, 2014 to May 10th, 2014, seven days in total. The dataset C covers 118,255,953 records produced by all of QQ users and 123,539,778 records produced by all of WeChat users in Chongqing China from Jul 17th, 2014 to Jul 23rd, 2014, seven days in total. The records consist of user id, traffic type, start time when users access to the server, end time and duration. In this paper, we focus on analyzing the temporal characteristics. The format of the dataset is shown in Table 1. Through data preprocessing, we can get the experimental datasets, including File A, File B and File C in S1 Supporting Information.

**Table 1:** Dataset format.

PHONE_NUMBER	TRAFFIC_TYPE	START_TIME / t
user 1	300	May.3,2014, 11:59:59.988604000 pm / t1
user 2	300	May.3,2014, 11:59:59.997355000 pm / t2
user 3	304	May.4,2014, 12:00:00.007422000 am / t3
user 4	300	May.4,2014, 12:00:00.017316000 am / t4
…	…	…

For analyzing temporal characteristics, we count the inter-event time distribution as follows:

1. We set the time window size as one day [23], that is, we use one day’s records to analyze temporal characteristics. The consecutive access times to the same server are denoted as *t*_*1*_, *t*_*2*_, …, *t*_*n*_, where *n* represents the number of record, *t*_*1*_ < *t*_*2*_ < … < *t*_*n*_. The inter-event time *τ* represents the time intervals between two consecutive access times as calculated below:
  $$\tau =t_{i+1}-t_{i}$$ (1)
2. In this paper, we analyze the distribution, *P*(*τ*) of the inter-event times *τ*. To be convenient for statistics, we use a term named *T* to calculate *P*(*τ*). $\begin{array}{l} \;\;P(\tau )=ae^{b\tau }\\ a=0.06921\\ b=-74.34 \end{array}$ is equal to the ratio of total number of *τ* which are in *((i* − 1)*T, *i* * *T)* which we call *n*_*i*_ and total number of records *N*,
  $$P(\tau =T_{i-1,i})=P_{i}=\frac{n_{i}}{N}$$ (2)
  where *i* represents the number of *T*.
3. We calculate the *P*(*τ*) when *T* is set to 0.001s, 0.1s and 1s respectively and find that only when *T* is 0.001s, do the inter-event time distributions of IM services follow a piecewise distribution. So it’s worth looking in more detail at the distributions when *T* is set to 0.001s.

## Methods

### Temporal characteristics of QQ users’ online behavior

We calculate P(τ) of QQ when *T* is 0.001s, 0.1s and 1s by Eq (2), and the results are shown in Figs 1–9.

**Fig 1:**
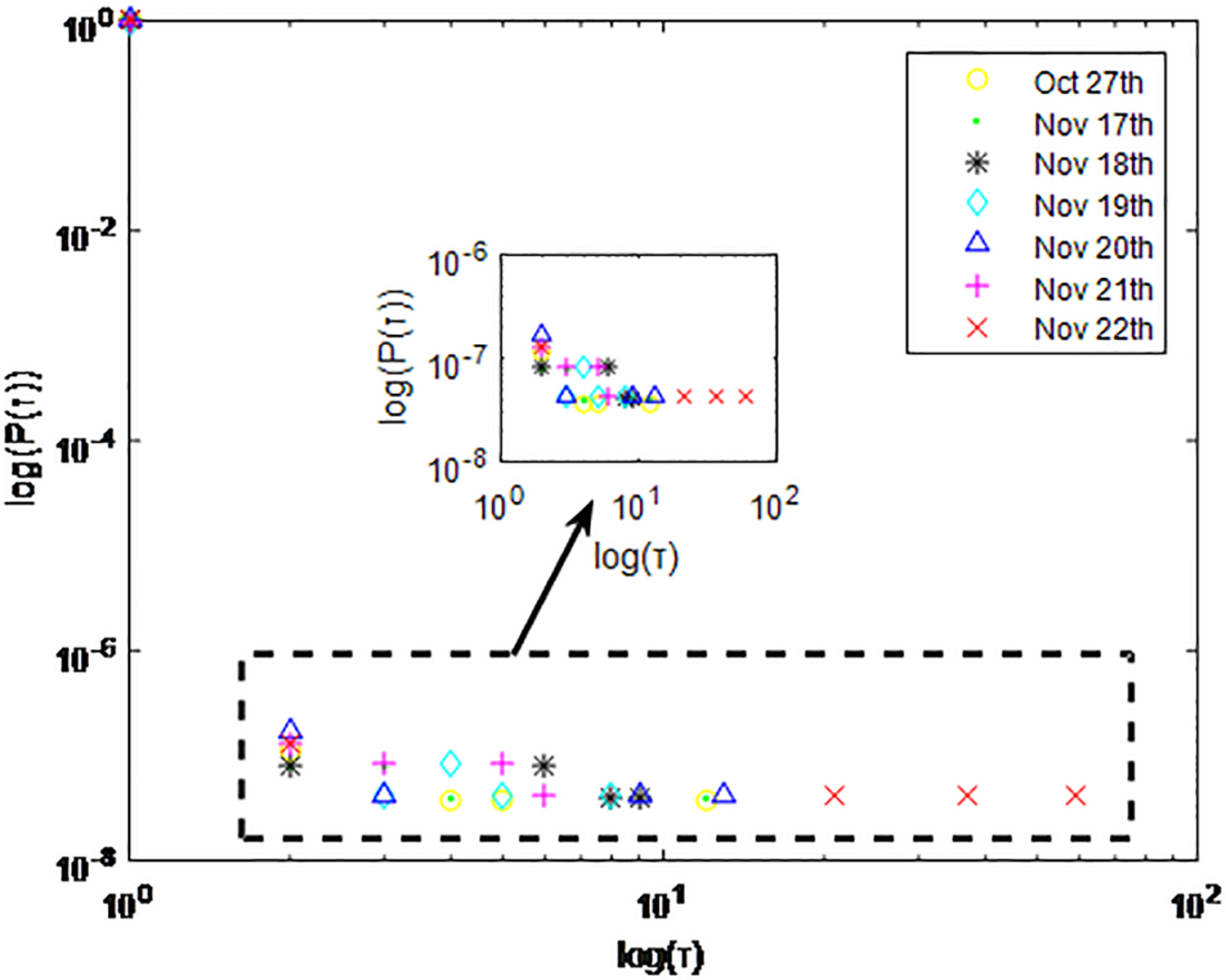
Distributions *P*(*τ*) of inter-event time *τ* for QQ in dataset A, *T* = 1*s*.

**Fig 2:**
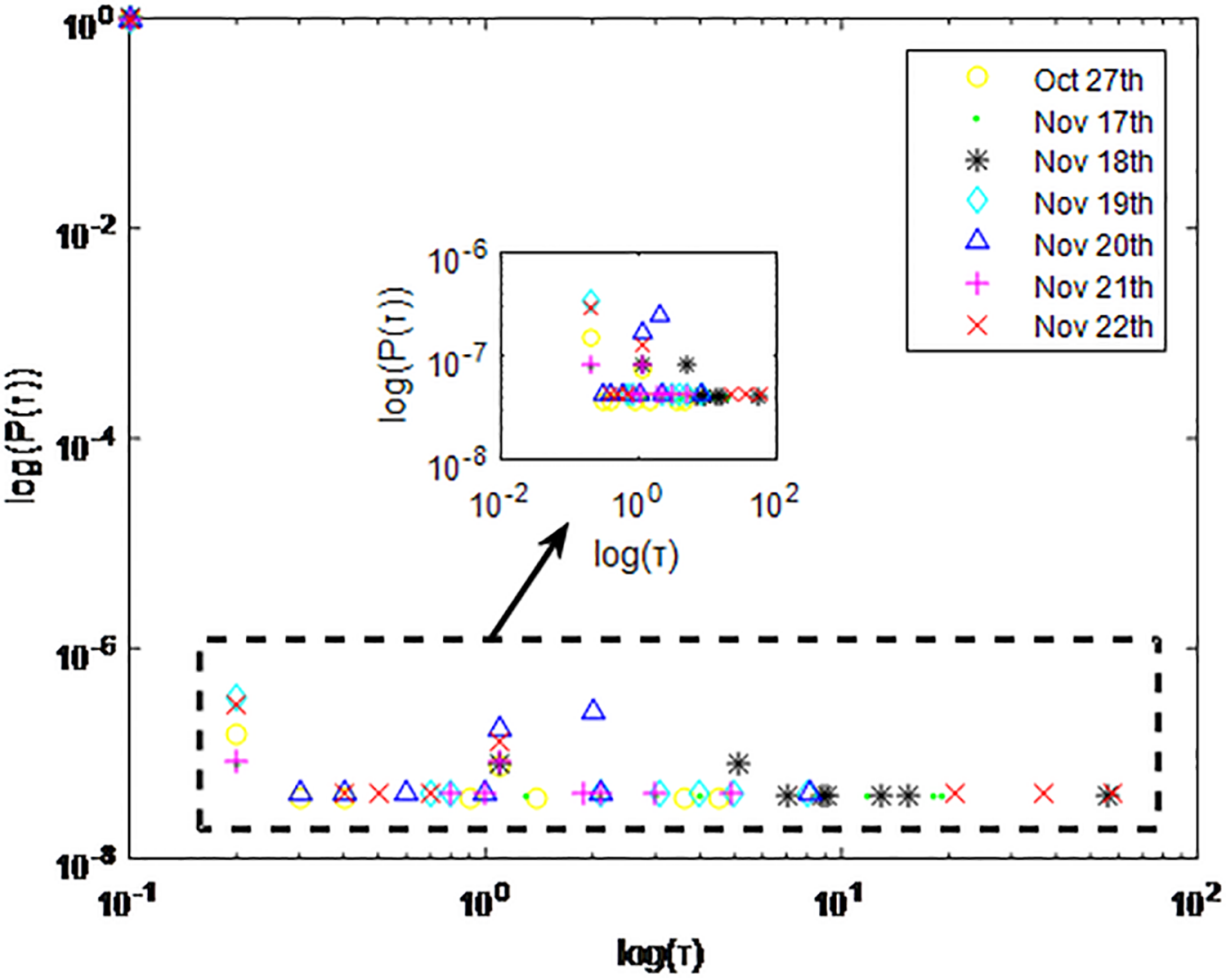
Distributions *P*(*τ*) of inter-event time *τ* for QQ dataset A, *T* = 0.1*s*.

**Fig 3:**
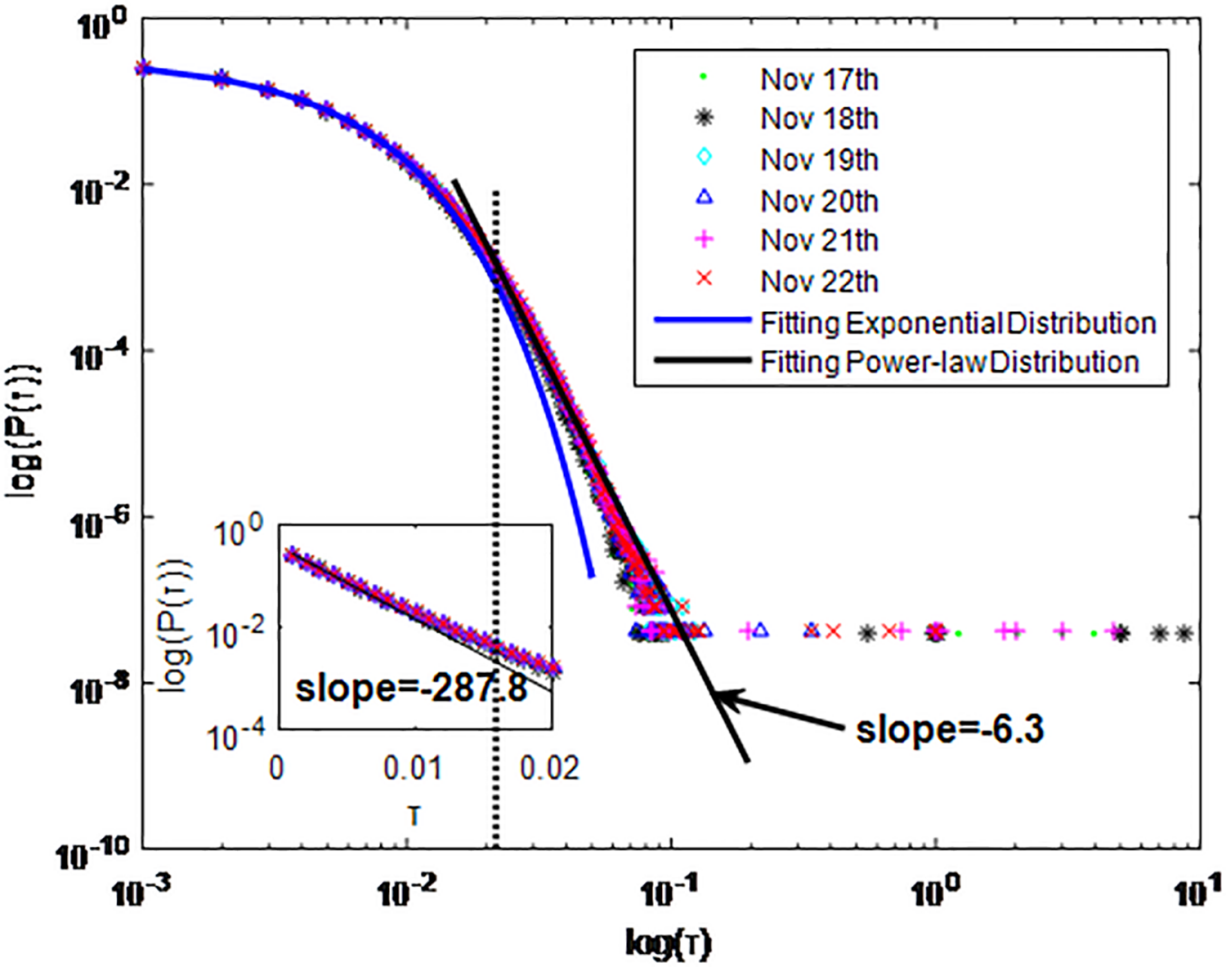
Distributions *P*(*τ*) of inter-event time *τ* for QQ in dataset A, *T* = 0.001*s*.

**Fig 4:**
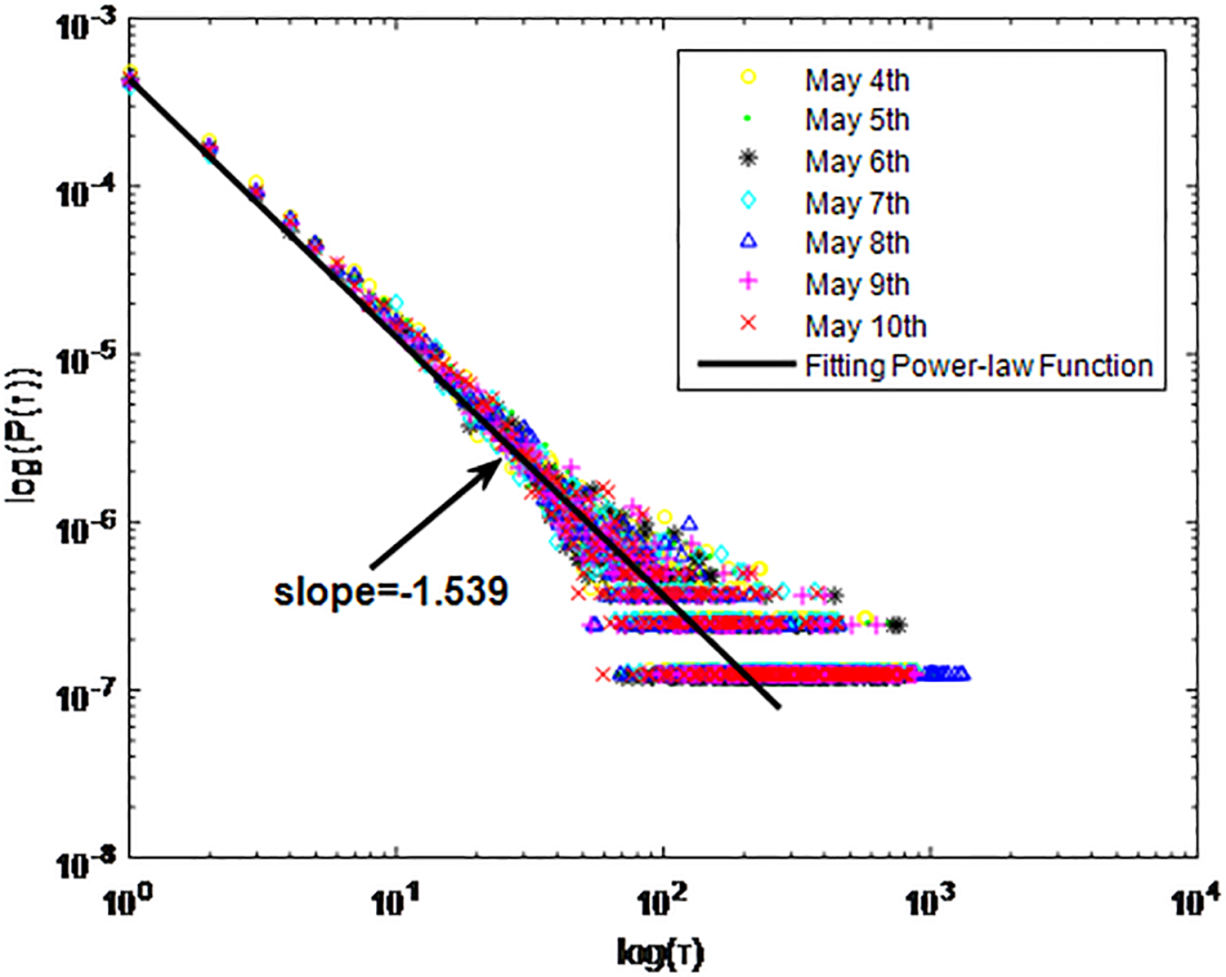
Distributions *P*(*τ*) of inter-event time *τ* for QQ in dataset B, *T* = 1*s*.

**Fig 5:**
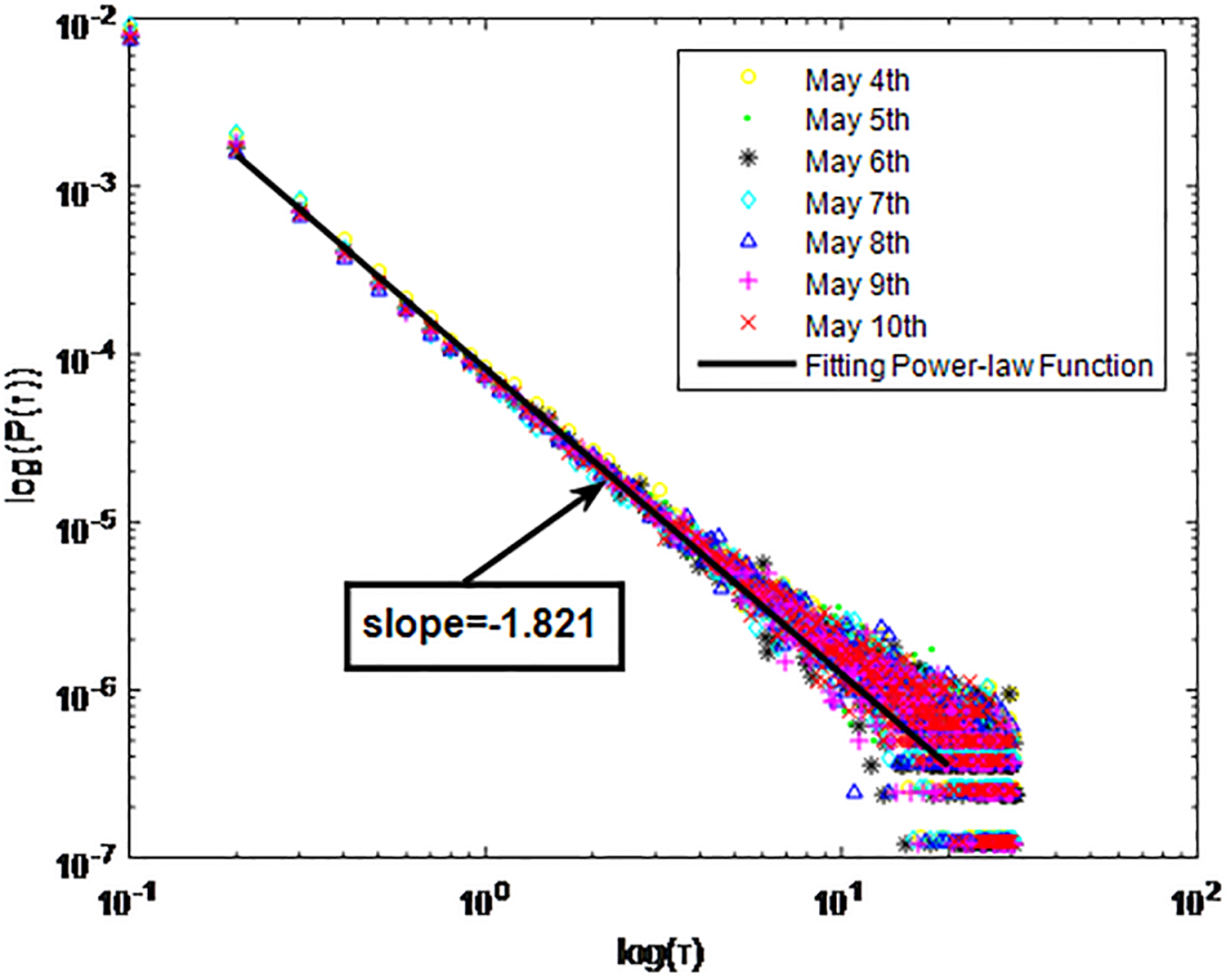
Distributions *P*(*τ*) of inter-event time *τ* for QQ in dataset B, *T* = 0.1*s*.

**Fig 6:**
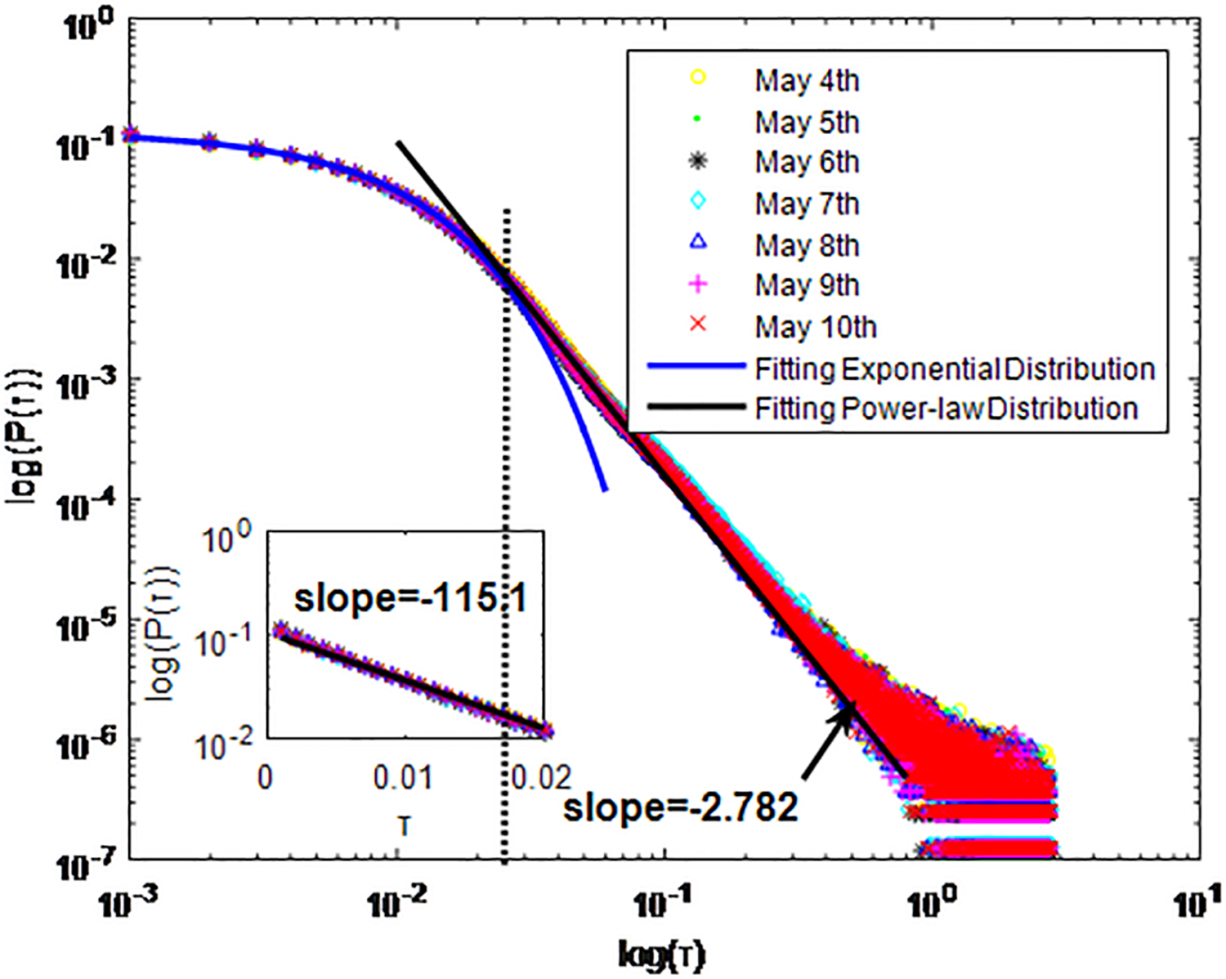
Distributions *P*(*τ*) of inter-event time *τ* for QQ in dataset B, *T* = 0.001*s*.

**Fig 7:**
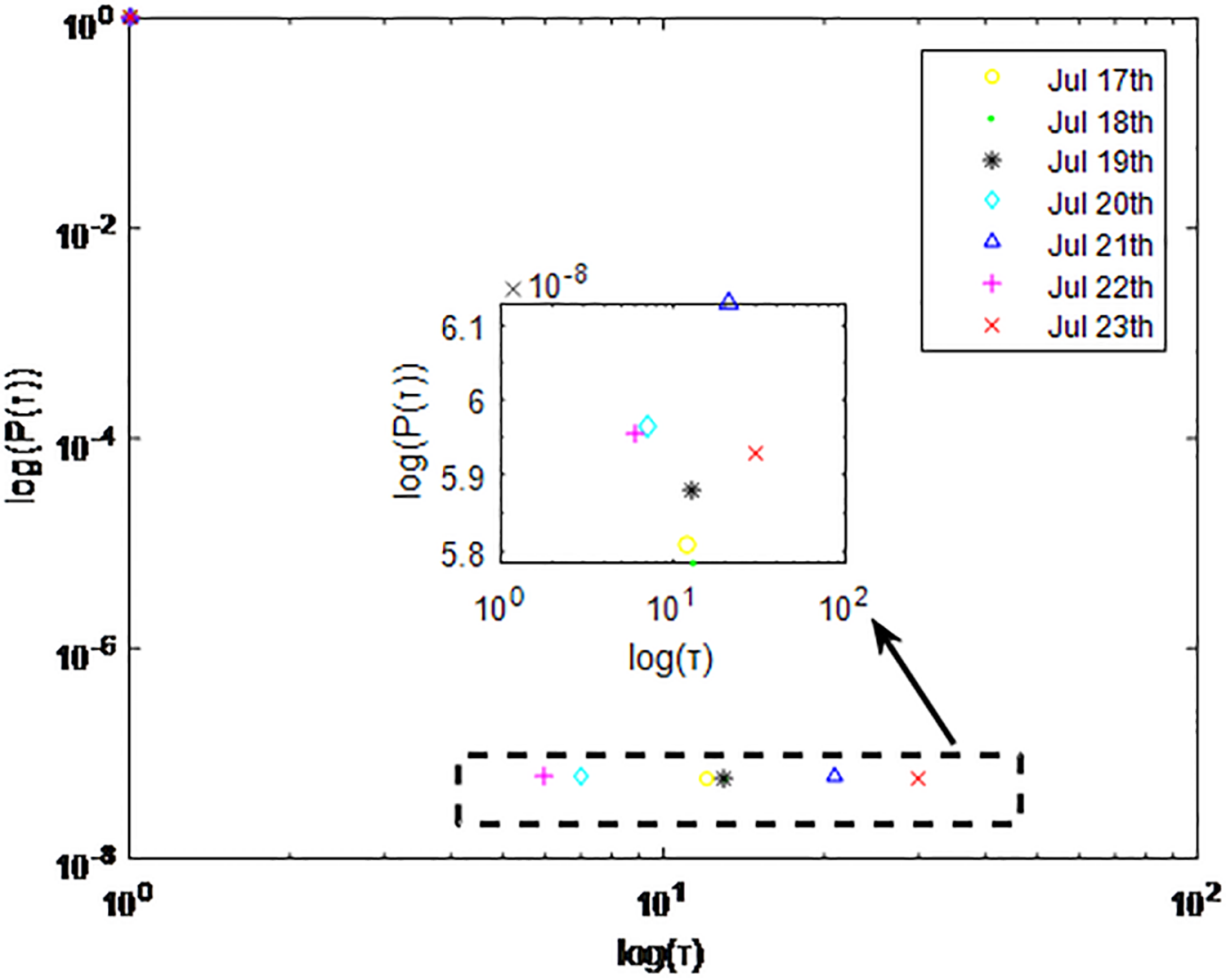
Distributions *P*(*τ*) of inter-event time *τ* for QQ in dataset C, *T* = 1*s*.

**Fig 8:**
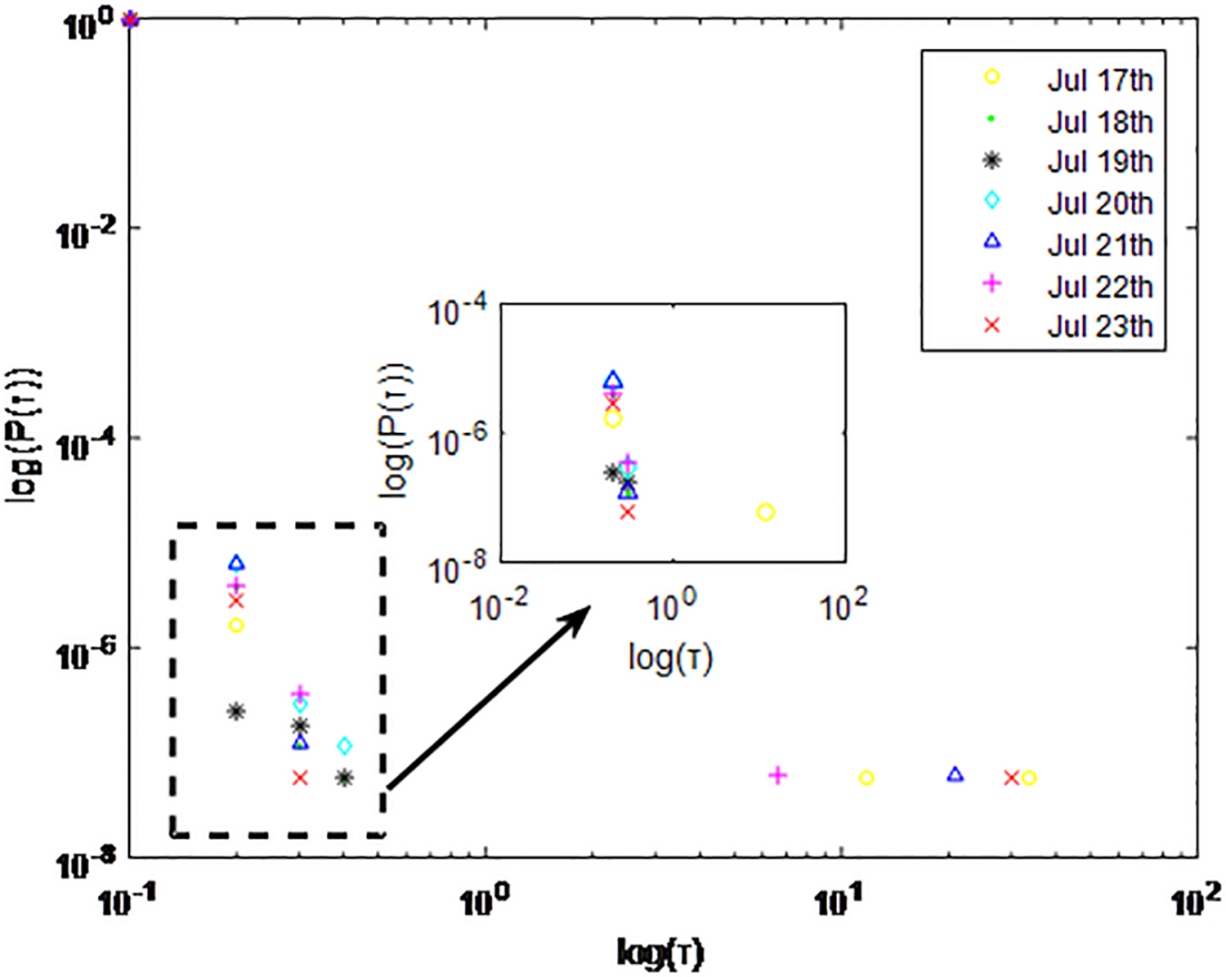
Distributions *P*(*τ*) of inter-event time *τ* for QQ in dataset C, *T* = 0.1*s*.

**Fig 9:**
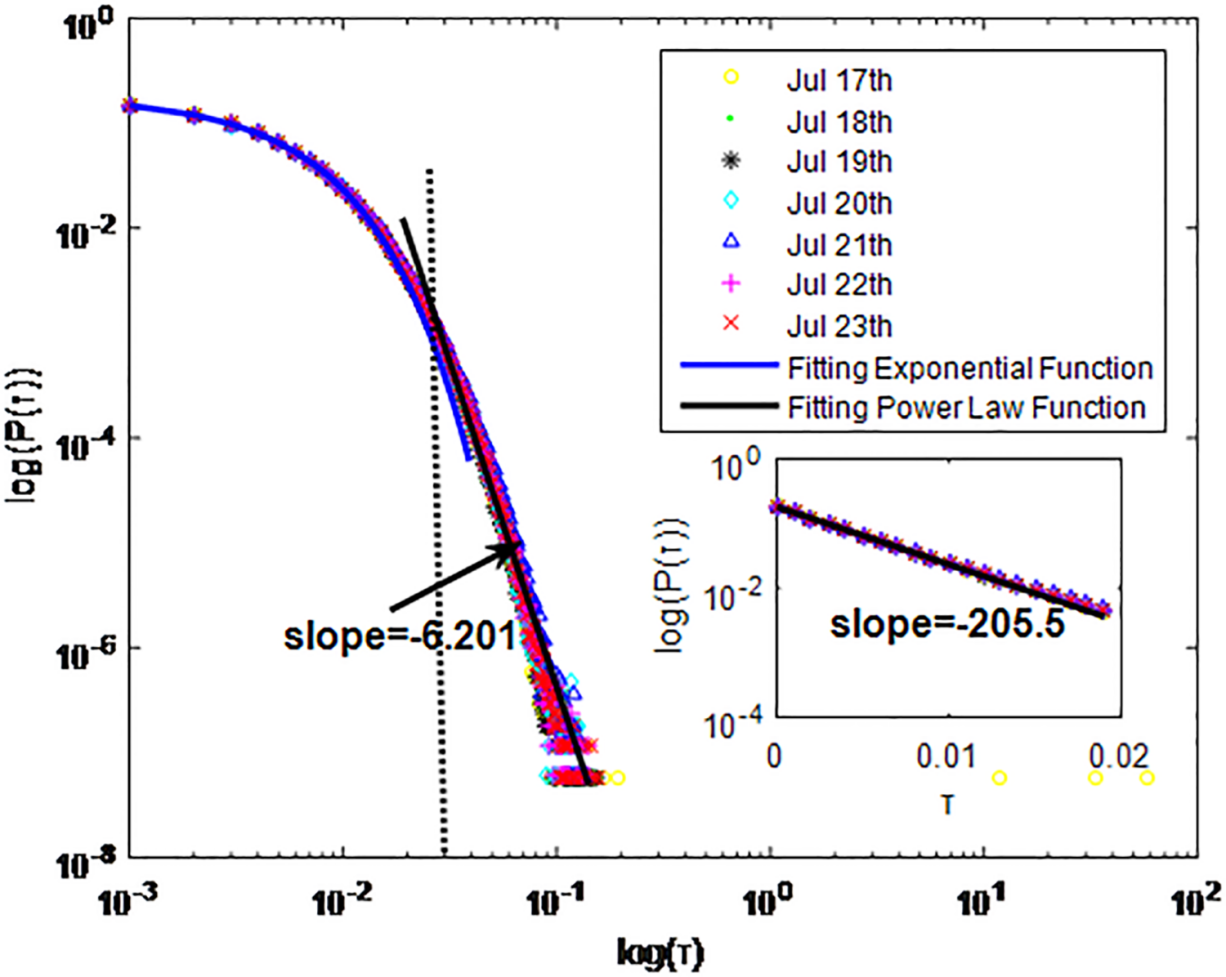
Distributions *P*(*τ*) of inter-event time *τ* for QQ in dataset C, *T* = 0.001*s*.

The X axis represents the logarithm of the inter-event time *τ*. The Y axis represents the logarithm of the distribution *P*(*τ*). Points with a different sign show *P*(*τ*) on different days. Figs 1, 2 and 3 are the distributions *P*(*τ*) in dataset A when *T* is 1s, 0.1s and 0.001s, respectively. Figs 4, 5 and 6 are the distributions *P*(*τ*) in dataset B when *T* is 1s, 0.1s and 0.001s, respectively. Figs 7, 8 and 9 are the distributions *P*(*τ*) in dataset C when *T* is 1s, 0.1s and 0.001s, respectively.

From Figs 3, 6 and 9 we can see that when *T* is 0.001s, *P*(*τ*) in both cities can be described by a piecewise distribution, the vertical dotted line marks the transition point of the piecewise distribution, indicating that the QQ users’ online behavior is heterogeneous in different time scales. This heterogeneous nature doesn’t emerge in other pictures. In Figs 1, 2, 7 and 8, most of *τ* are smaller than 0.1s, so *P*(*τ*) converges to a point. The inset shows the distribution of *τ* > 0.1*s*. In Figs 4 and 5, *P*(*τ*) follows a power-law distribution when *T* is 0.1s and 1s. In this paper, we focus on the situation when *T* is 0.001s. We set *τ*_*0*_ as the transition point of the piecewise distribution and *τ*_*0*_ is the closest point between the exponential function and the power-law distribution.

From Figs 3, 6 and 9 we can see that the trend of *P*(*τ*) in different days is quite similar. All of them fit piecewise distribution. When *τ* < *τ*_*0*_, the distribution function is *P*(*τ*) = *ae*^*bτ*^, where the exponent parameter *b* is 287.8, 115.1 and 205.5 respectively. When *τ* > *τ*_*0*_, the distribution function is *P*(*τ*) = *aτ*^*b*^, where the power-law exponent parameter *b* is 6.3, 2.782 and 6.201 respectively. We use the correlation coefficient to evaluate the goodness of fit. The value of *R*^2^ shown in Table 2 is defined as below.

$$R^{2}=\frac{\sum_{i=1}^{n}w_{i}{\left({\hat{y}}_{i}-{\bar{y}}_{i}\right)}^{2}}{\sum_{i=1}^{n}w_{i}{\left(y_{i}-{\bar{y}}_{i}\right)}^{2}}$$ (3)

**Table 2:** Fitting functions of inter-event time distribution.

Service	Dataset	*T*	Bimodal Distribution?		Parameters of Fitting Function	Goodness of Fit *R*^2^
**QQ**	A	0.001s	Yes	*τ* < *τ*_*0*_	Exponential: $\begin{array}{l} P(\tau )=ae^{b\tau }\\ a=0.3298\\ b=-287.8 \end{array}$	0.9998
				*τ* > *τ*_*0*_	power-law tail: $\begin{array}{l} P(\tau )=a\tau^{b}\\ a=3.696*e^{-14}\\ b=-6.3 \end{array}$	0.9917
**QQ**	B	0.001s	Yes	*τ* < *τ*_*0*_	Exponential: $\begin{array}{l} \;P(\tau )=ae^{b\tau }\\ a=0.1158\\ b=-115.1 \end{array}$	0.9986
				*τ* > *τ*_*0*_	power-law tail: $\begin{array}{l} P(\tau )=a\tau^{b}\\ a=2.592*e^{-7}\\ b=-2.782 \end{array}$	0.9994
**QQ**	C	0.001s	Yes	*τ* < *τ*_*0*_	Exponential: $\begin{array}{l} \;P(\tau )=ae^{b\tau }\\ a=0.1833\\ b=-205.5 \end{array}$	0.9998
				*τ* > *τ*_*0*_	power-law tail: $\begin{array}{l} P(\tau )=a\tau^{b}\\ a=2.662*e^{-13}\\ b=-6.201 \end{array}$	0.99
**WeChat**	A	0.001s	Yes	*τ* < *τ*_*0*_	Exponential: $\begin{array}{l} \;P(\tau )=ae^{b\tau }\\ a=0.06921\\ b=-74.34 \end{array}$	0.9996
				*τ* > *τ*_*0*_	power-law: $\begin{array}{l} P(\tau )=a\tau^{b}\\ a=6.656*e^{-7}\\ b=-2.68 \end{array}$	0.9984
**WeChat**	B	0.001s	Yes	*τ* < *τ*_*0*_	Exponential: $\begin{array}{l} P(\tau )=ae^{b\tau }\\ a=1.272\\ b=-847.2 \end{array}$	0.9988
				*τ* > *τ*_*0*_	power-law: $\begin{array}{l} P(\tau )=a\tau^{b}\\ a=8.565*e^{-8}\\ b=-2.228 \end{array}$	0.9955
**WeChat**	C	0.001s	Yes	*τ* < *τ*_*0*_	Exponential: $\begin{array}{l} P(\tau )=ae^{b\tau }\\ a=0.1986\\ b=-228 \end{array}$	0.9993
				*τ* > *τ*_*0*_	power-law: $\begin{array}{l} P(\tau )=a\tau^{b}\\ a=2.691*e^{-10}\\ b=-4.19 \end{array}$	0.9917

In this paper, *R*^2^ can be calculated by the average value of real data in seven days and the fitting data in Fig 1.

It is worth noting that the tail of *P*(*τ*) is fat in Fig 1(d), 1(e) and 1(f). That is because the tail of *P*(*τ*) changes too much. Here, to find whether there are other functions to fit the fat tail better, so we do an experiment as follows. In Fig 1, the tail of *P*(*τ*) belongs to heavy-tailed distribution. Moreover, the Gaussian and Weibull are two of the most popular heavy-tailed distributions. Furthermore, we use Gaussian and Weibull distribution to fit the tail of *P*(*τ*) respectively and analyze their goodness of fit. The value of *R*^2^ is shown in Table 3. We find *R*^2^ of them are less than 0.5, so both of the fitting functions can not fit *P*(*τ*)better than a power-law distribution.

**Table 3:** The value of *R*^2^ when *P*(*τ*) fits Gaussian and Weibull for dataset B.

Service	Function	*R*^2^(*T* = 1*s*)	*R*^2^(*T* = 0.1*s*)	*R*^2^(*T* = 0.001*s*)
**QQ**	Gaussian $P(\tau )=ae^{-{\left((\tau -b)/c\right)}^{2}}$	Can’t fit	0.3185	0.2474
	Weibull $P(\tau )=ab\tau^{b-1}e^{-a\tau^{b}}$	-6312	-0.4634	-845.1
**WeChat**	Gaussian $P(\tau )=ae^{-{\left((\tau -b)/c\right)}^{2}}$	0.3618	0.397	0.4699
	Weibull $P(\tau )=ab\tau^{b-1}e^{-a\tau^{b}}$	-49.88	-1.134	-3312

From Table 2, we find that the parameter *b* of two cities are different for QQ. The more popular service, the bigger *b* is. The specific analysis process is introduced in part V.

### Temporal characteristics of WeChat users’ online behavior

We calculate P(τ) of WeChat when *T* is 0.001s, 0.1s and 1s by Eq (2), and the results are shown in Figs 10–18.

**Fig 10:**
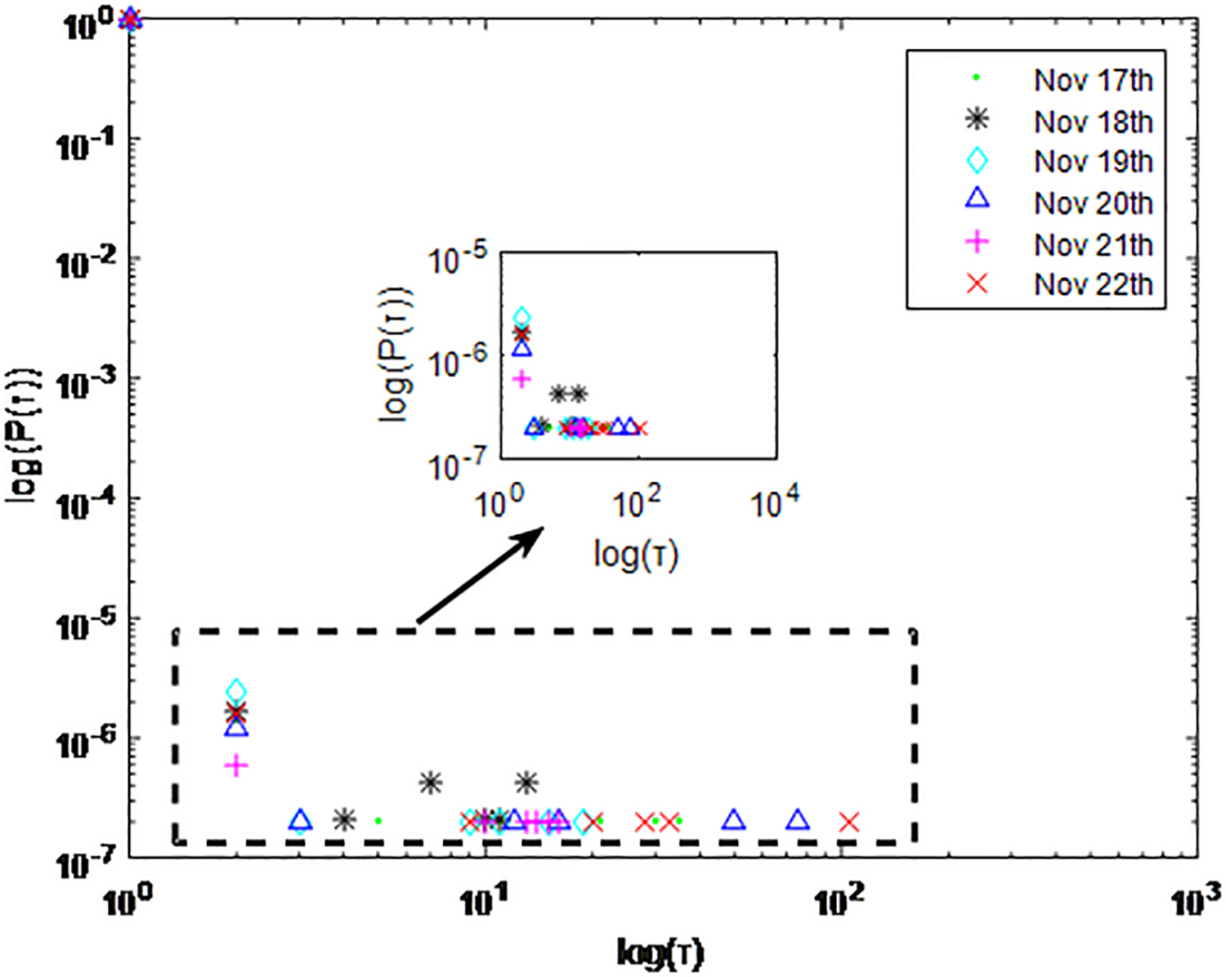
Distributions *P*(*τ*) of inter-event time *τ* for WeChat in dataset A, *T* = 1*s*.

**Fig 11:**
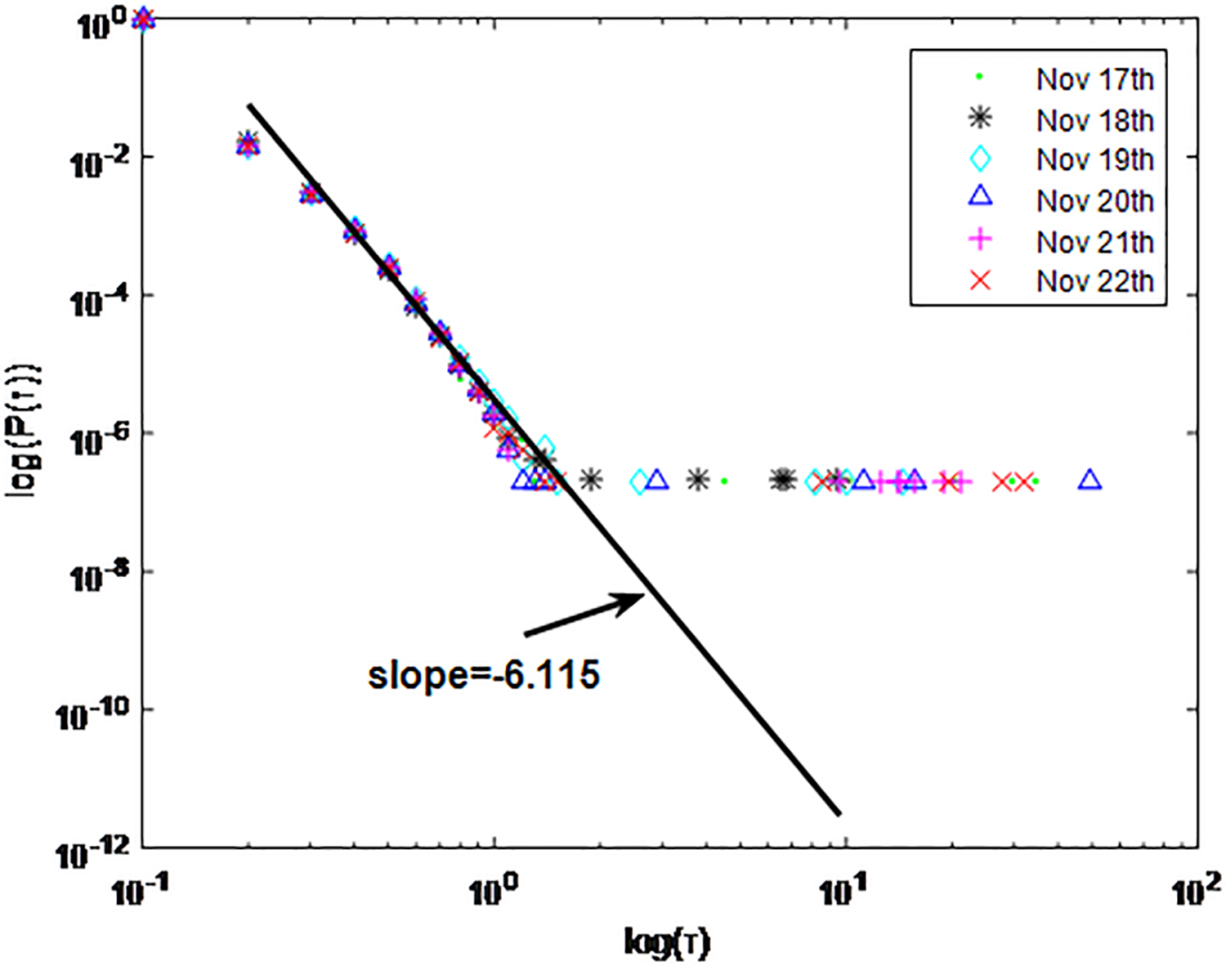
Distributions *P*(*τ*) of inter-event time *τ* for WeChat in dataset A, *T* = 0.1*s*.

**Fig 12:**
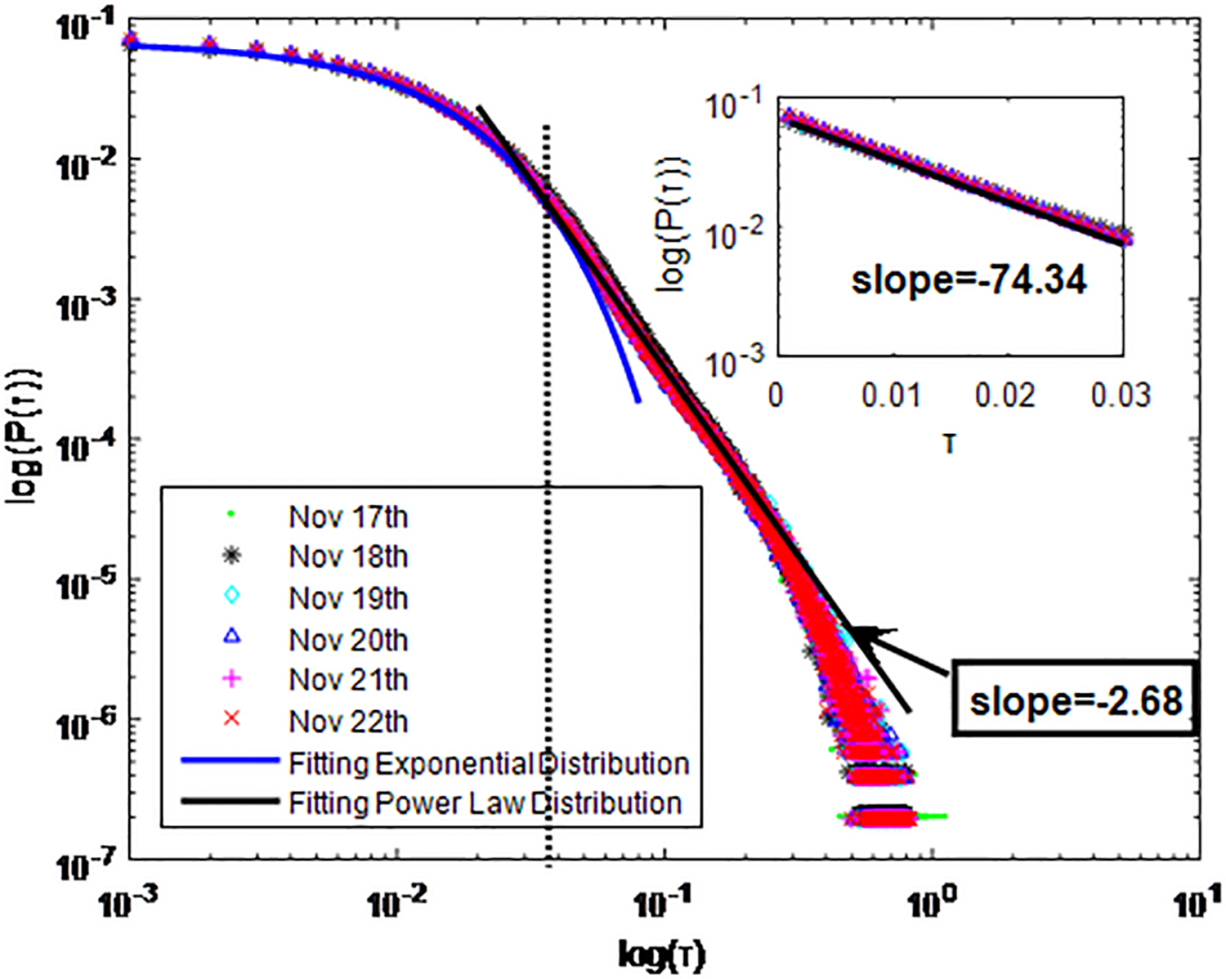
Distributions *P*(*τ*) of inter-event time *τ* for WeChat in dataset A, *T* = 0.001*s*.

**Fig 13:**
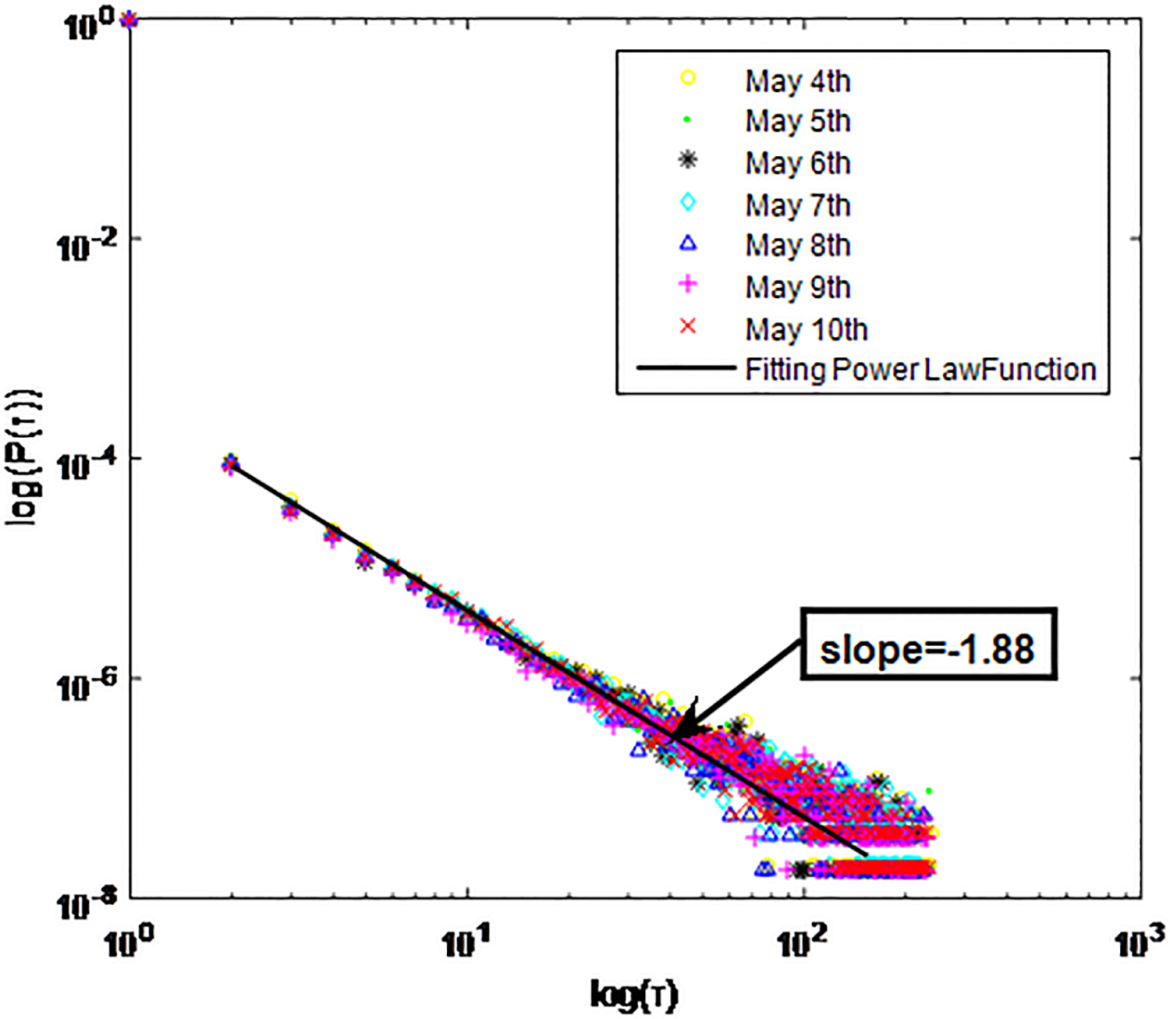
Distributions *P*(*τ*) of inter-event time *τ* for WeChat in dataset B, *T* = 1*s*.

**Fig 14:**
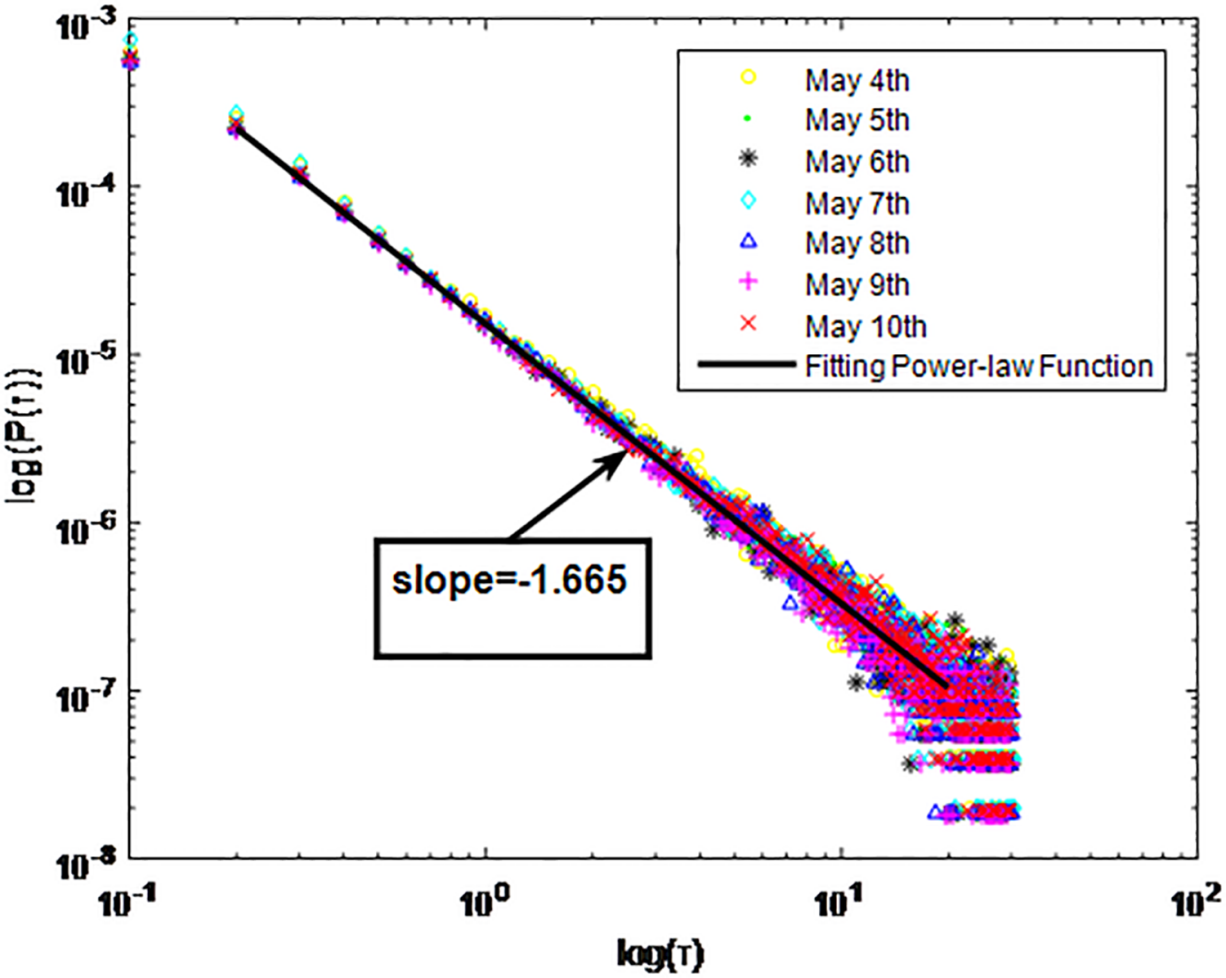
Distributions *P*(*τ*) of inter-event time *τ* for WeChat in dataset B, *T* = 0.1*s*.

**Fig 15:**
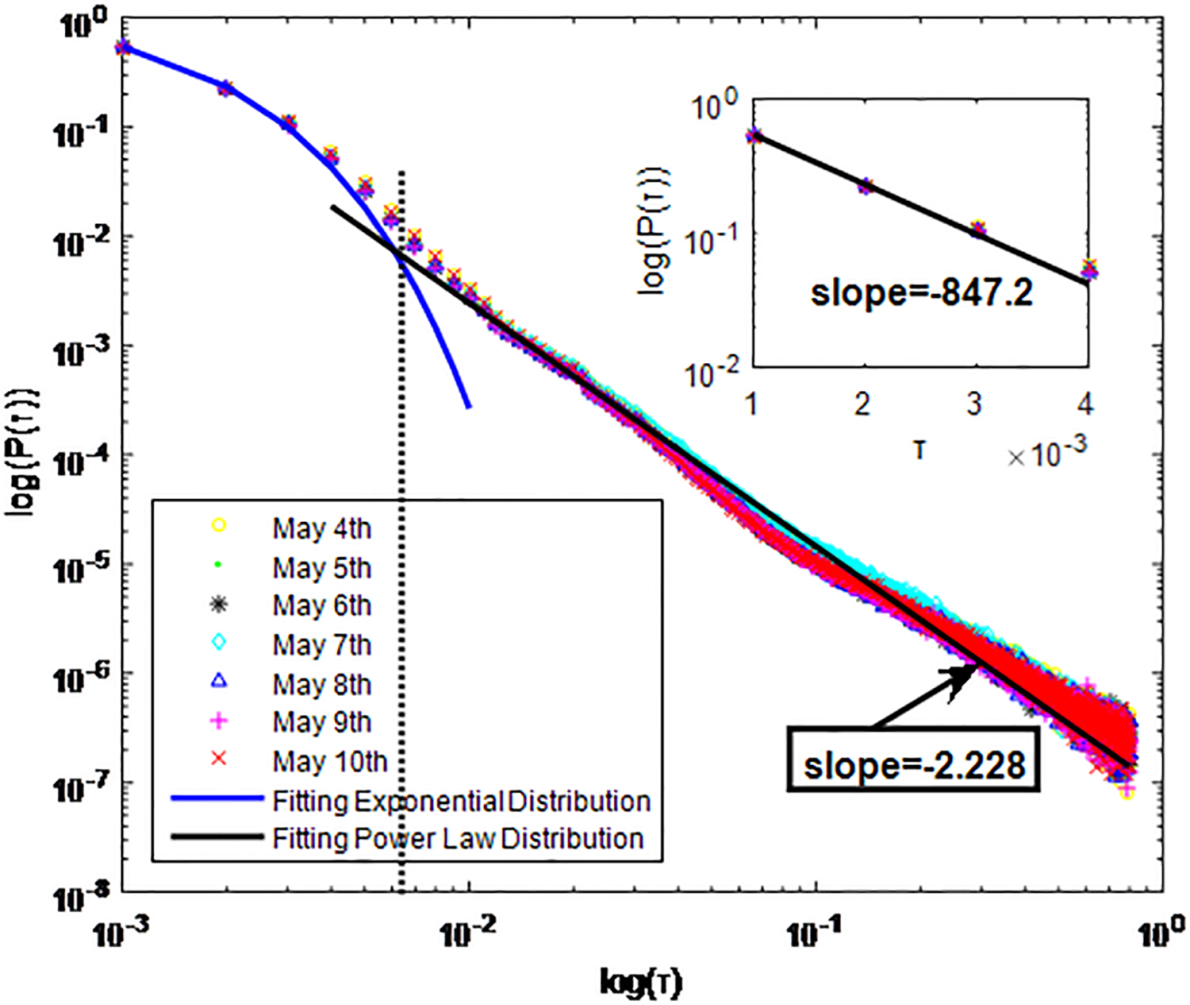
Distributions *P*(*τ*) of inter-event time *τ* for WeChat in dataset B, *T* = 0.001*s*.

**Fig 16:**
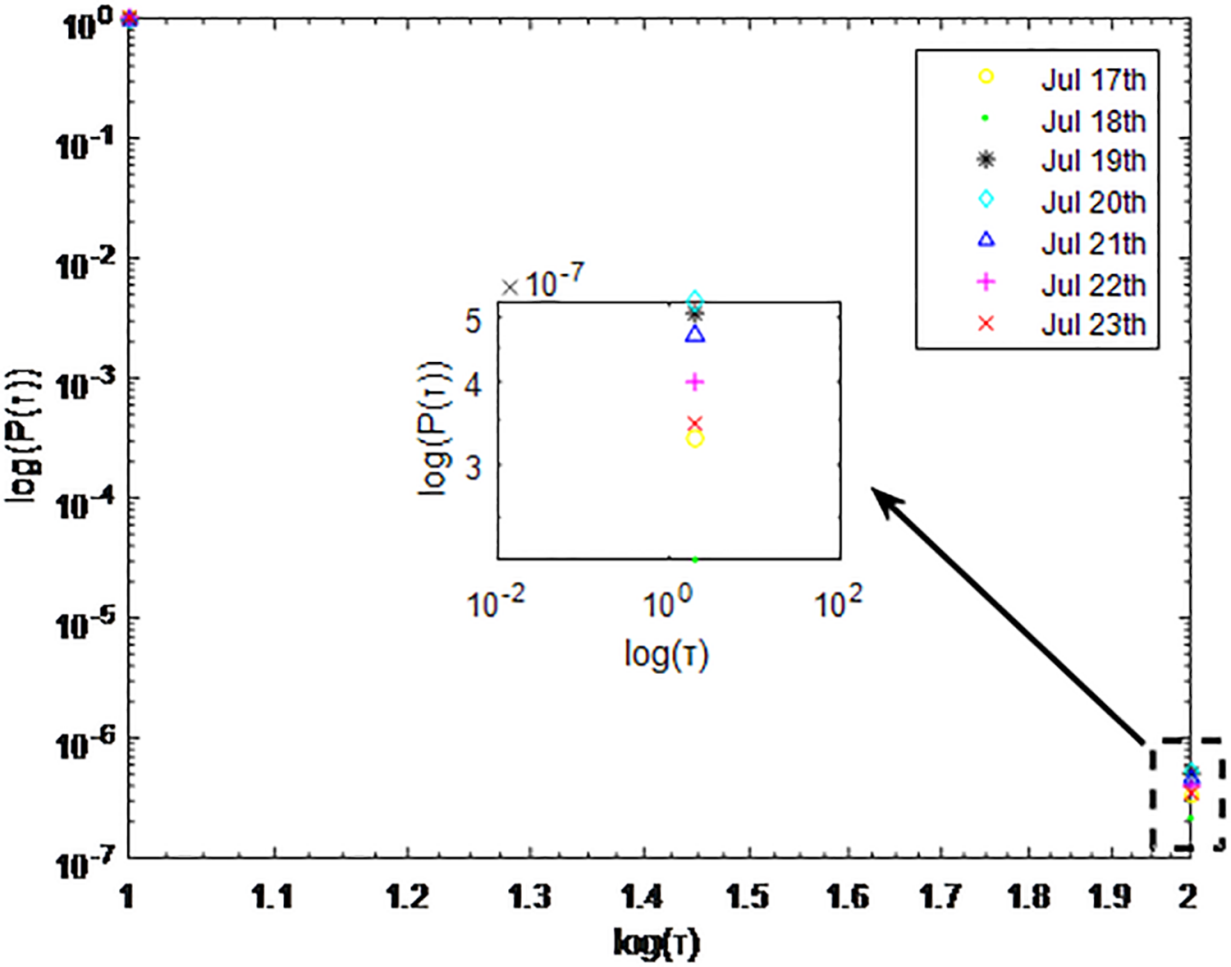
Distributions *P*(*τ*) of inter-event time *τ* for WeChat in dataset C, *T* = 1*s*.

**Fig 17:**
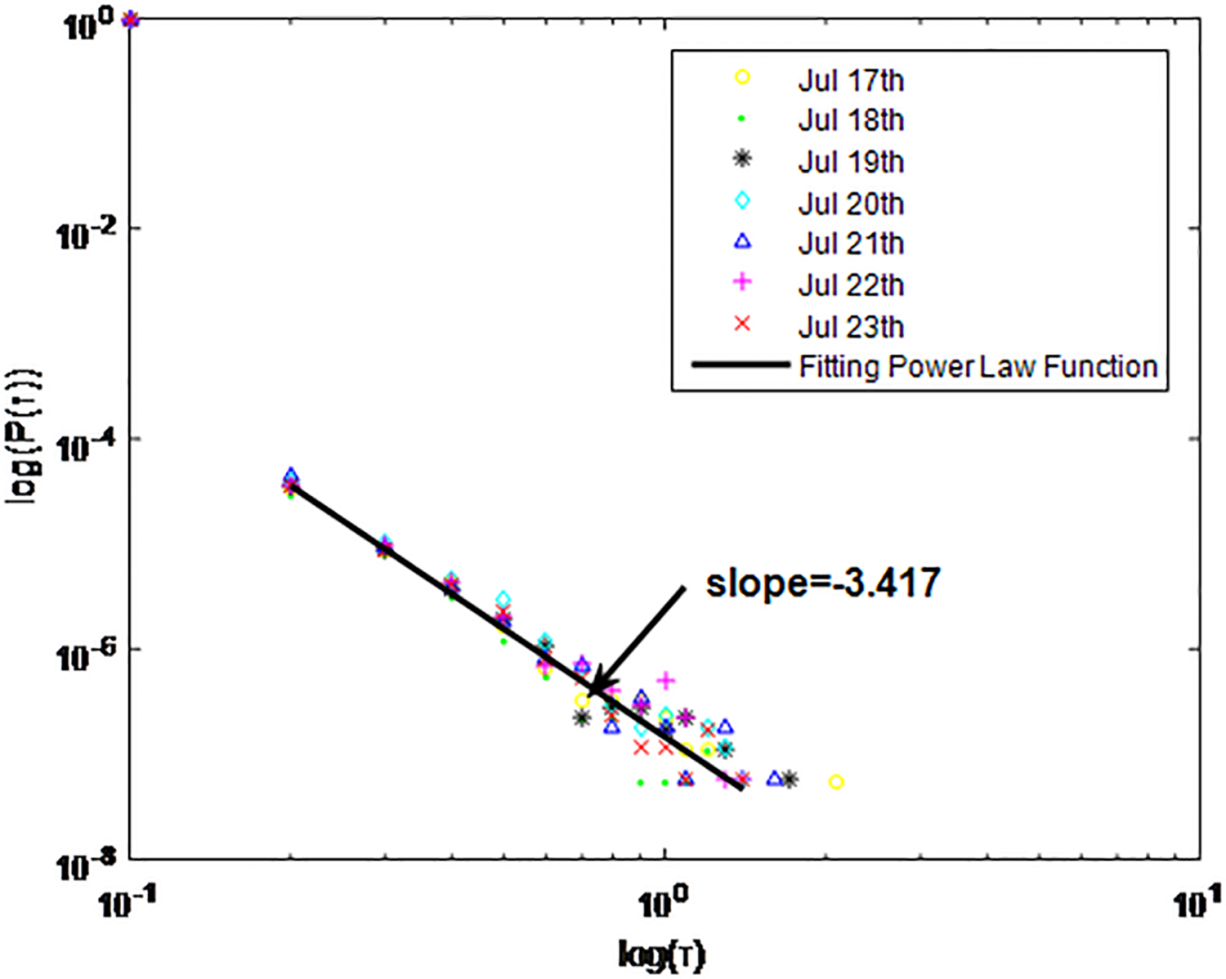
Distributions *P*(*τ*) of inter-event time *τ* for WeChat in dataset C, *T* = 0.1*s*.

**Fig 18:**
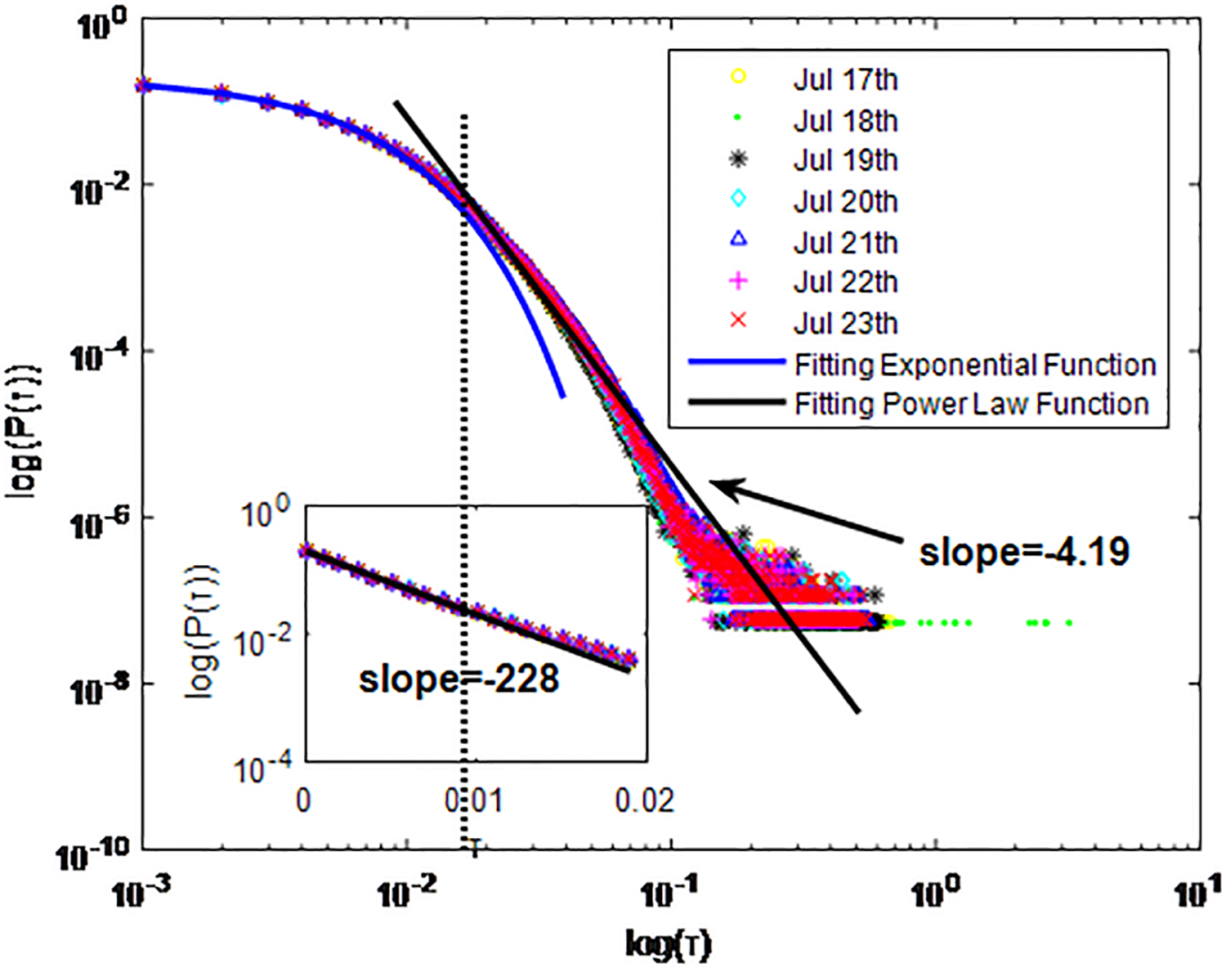
Distributions *P*(*τ*) of inter-event time *τ* for WeChat in dataset C, *T* = 0.001*s*.

The X axis represents the logarithm of the inter-event time *τ*. The Y axis represents the logarithm of the distribution *P*(*τ*). Dots with a different color show *P*(*τ*) on different days. Figs 10, 11 and 12 are the distributions *P*(*τ*) for dataset A when *T* is 1s, 0.1s and 0.001, respectively. Figs 13, 14 and 15 are the distributions *P*(*τ*) for dataset B when *T* is 1s, 0.1s and 0.001s, respectively. Figs 16, 17 and 18 are the distributions *P*(*τ*) for dataset C when *T* is 1s, 0.1s and 0.001s.

WeChat has the same communication mechanism as QQ, which also can be seen from Figs 10–18. Only one different situation is that when *T* = 0.1*s*, *P*(*τ*) of WeChat in dataset A and dataset C fit power-law distribution. *P*(*τ*) of WeChat in both cities also follow a piecewise distribution when the *T* is 0.001s. In Figs 12, 15 and 18 the exponent parameter of *P*(*τ*) is 74.34, 847.2 and 228 respectively when *τ* < *τ*_*0*_. The power-law exponent parameter of *P*(*τ*) for City-A and City-B is 2.68, 2.228 and 4.19 respectively when *τ* > *τ*_*0*_. The specific fitting conditions can be seen in Table 2. The tail of *P*(*τ*) is fat in Figs 13, 14 and 15 which is similar to Figs 1–9. We also use Gaussian and Weibull distribution to fit them. The value of *R*^2^ is shown in Table 3.

### Relationship between inter-event time distribution and popularity of services

To explore the reason why the parameter *b* is different in different cities, we count the number of records produced by IM services users as shown in Table 4. For QQ, no matter whether *τ* < *τ*_*0*_ or *τ* > *τ*_*0*_, the larger the size of records, the bigger the exponent parameter of fitting function. For WeChat, it has the same rules with QQ when *τ* < *τ*_*0*_. But when *τ* > *τ*_*0*_, the rules only exists in the same city. The size of records can show the popularity of a service in the city. So there is some relationship between inter-event time distribution and popularity of services, which may be related to the urban economic level.

**Table 4:** Comparison of data in different cities.

		QQ		WeChat	
Dataset	Date	The number of records	Parameters of fitting functions	The number of records	Parameters of fitting functions
**A**	Nov.17	25,425,622	*τ* < *τ*_*0*_ Exponential: $\begin{array}{l} P(\tau )=ae^{b\tau }\\ a=0.3298\\ b=-287.8 \end{array}$ *τ* > *τ*_*0*_ power-law: $\begin{array}{l} P(\tau )=a\tau^{b}\\ a=3.696*e^{-14}\\ b=-6.3 \end{array}$	4,902,654	*τ* < *τ*_*0*_ Exponential: $\begin{array}{l} \;P(\tau )=ae^{b\tau }\\ a=0.06921\\ b=-74.34 \end{array}$ *τ* > *τ*_*0*_ power-law $\begin{array}{l} P(\tau )=a\tau^{b}\\ a=6.656*e^{-7}\\ b=-2.68 \end{array}$
	Nov.18	24,768,721		4,803,353	
	Nov.19	23,674,143		5,051,712	
	Nov.20	23,705,937		5,113,650	
	Nov.21	23,668,333		5,092,997	
	Nov.22	23,649,742		5,106,358	
**B**	May.4	7,537,646	*τ* < *τ*_*0*_ Exponential: $\begin{array}{l} \;P(\tau )=ae^{b\tau }\\ a=0.1158\\ b=-115.1 \end{array}$ *τ* > *τ*_*0*_ power-law $\begin{array}{l} P(\tau )=a\tau^{b}\\ a=2.592*e^{-7}\\ b=-2.782 \end{array}$	49,247,002	*τ* < *τ*_*0*_ Exponential: $\begin{array}{l} P(\tau )=ae^{b\tau }\\ a=1.272\\ b=-847.2 \end{array}$ *τ* > *τ*_*0*_ power-law: $\begin{array}{l} P(\tau )=a\tau^{b}\\ a=8.565*e^{-8}\\ b=-2.228 \end{array}$
	May.5	7,994,897		52,345,169	
	May.6	8,343,569		54,084,772	
	May.7	7,725,910		50,371,562	
	May.8	8,123,377		53,857,018	
	May.9	8,181,384		55,113,740	
	May.10	8,035,997		51,268,788	
**C**	Jul 17	17,213,743	*τ* < *τ*_*0*_ Exponential: $\begin{array}{l} \;P(\tau )=ae^{b\tau }\\ a=0.1833\\ b=-205.5 \end{array}$ *τ* > *τ*_*0*_ power-law: $\begin{array}{l} P(\tau )=a\tau^{b}\\ a=2.662*e^{-13}\\ b=-6.201 \end{array}$	18,314,393	*τ* < *τ*_*0*_ Exponential: $\begin{array}{l} P(\tau )=ae^{b\tau }\\ a=0.1986\\ b=-228 \end{array}$ *τ* > *τ*_*0*_ power-law: $\begin{array}{l} P(\tau )=a\tau^{b}\\ a=2.691*e^{-10}\\ b=-4.19 \end{array}$
	Jul 18	17,286,189		18,516,738	
	Jul 19	17,006,725		17,700,355	
	Jul 20	16,770,599		17,098,411	
	Jul 21	16,316,213		17,040,162	
	Jul 22	16,796,260		17,481,080	
	Jul 23	16,866,224		17,388,639	

## Model

A variety of models have been proposed to explain the Non-Poisson characteristics of human behavior. Because of the heterogeneous character of IM services users’ behavior in different time scales, we don’t use any model put forward before.

The results obtained by real data show that when *τ* > *τ*_*0*_ the inter-event time distribution of IM services fits a power-law distribution and the exponents of the distributions are larger than 1. The new model proposed by Chengxu Wang et al. in [9], used the interest model to explain the power-law distribution in the large time scale because the exponent of it is larger than 1. And SHANG M S et al. hold that the interest model is also suitable for IM services [14], because users will visit the services once again based on their preference. So we consider the interest model to explain the power-law distribution of our results at first. The communication mechanism of IM services is the same as Short Messages Services[16]: Users start communications randomly, and the arrival of communications follows an Poisson process so that the inter-event time distribution follows exponential distribution. After triggering a communication, there are frequent exchanges of information between the user pairs. During this stage, the time interval is not uniform, the long waiting time exists and the inter-event time distribution follows a power-law distribution.

Based on the inter-event time distribution obtained by real data and the communication mechanism of IM services, in the paper, we consider a combined model of exponential function and interest model based on our analysis. For the case of *τ* < *τ*_*0*_, IM services users’ behavior is driven by a Poisson process. And when *τ* > *τ*_*0*_, the interest mechanism drives the behavior of IM users as referred to in [14].

### Exponent model

When *τ* < *τ*_*0*_, the arrival of events is a Poisson process. For the Poisson process, the inter-event time distribution follows an exponential distribution:

$$P_{p}(\tau )=\lambda e^{-\lambda \tau }$$ (4)

where *λ* is the arrival rate of events and *τ* is the inter-event time.

From Table 2 we can see that two parameters of the exponential function are different when *τ* < *τ*_*0*_, so in this paper we use the deduction form of exponential distribution called exponential model in this paper to fit the real data.

$$P_{p}(\tau )=\beta_{1}\lambda e^{-\lambda \tau }$$ (5)

where *β*_1_*λ* is equal to *a* in Table 2, and *λ* is equal to *b* in Table 2.

### Interest model

When *τ* > *τ*_*0*_, we use the interest model introduced in [11] to describe the inter-event time distribution. The interest model assumes that (i) the interest *x*_*i*_*(t)* at time step *t* of a user is quantified by the probability that an action will occur in this time; (ii) at each time step *t*, if an action occurs then the interest *x*_*i*_*(t)* is reset to 1; (iii) when the last action occurred at step *t*_0_, then the interest at step *t* is set as:

$$x_{i}(t)=\frac{1}{1+\alpha (t-t_{0})}$$ (6)

where *α* is a free parameter. If an action occurs at a certain time step *t*, the probability that next action occurs at time step *t* + Δ*t* is

$$P_{i}(\tau )∼\frac{1}{\alpha }\tau^{-(1+\frac{1}{\alpha })}$$ (7)

In the interest model,

$$\gamma =1+\frac{1}{\alpha }$$ (8)

where *γ* is the power exponent of the interest model. We can get *γ* from real data and evaluate *α* by Eq (8).

### Combination model

To get the combination model used in this paper, the exponential model and interest model should be combined. However, the Eqs (5) and (7), which represent the exponential model and interest model respectively, can’t be combined directly now. We rescale the interest model as:

$$x_{i}(t)=\frac{\beta_{2}}{1+\alpha (t-t_{0})}$$ (9)

where *β*_2_ is a tuning parameter which set the initial probability of the interest model. We adjust the value of *β*_2_ to fit the real data. Since *P*_*p*_(*τ*_0_) = *P*_*i*_(*τ*_0_), then

$$\beta_{{}_{1}}=\frac{\beta_{2}\frac{1}{\alpha }\tau_{0}{}^{-(1+\frac{1}{\alpha })}}{\lambda e^{-\lambda \tau_{0}}}$$ (10)

Finally, the combination model can be described as

$$\left\{ \begin{array}{l} P_{p}(\tau )=\beta_{1}\lambda e^{-\lambda \tau },\tau <\tau_{0}\\ P_{i}(\tau )∼\frac{\beta_{2}}{\alpha }\tau^{-(1+\frac{1}{\alpha })},\tau >\tau_{0} \end{array}\right.$$ (11)

**Fig 19:**
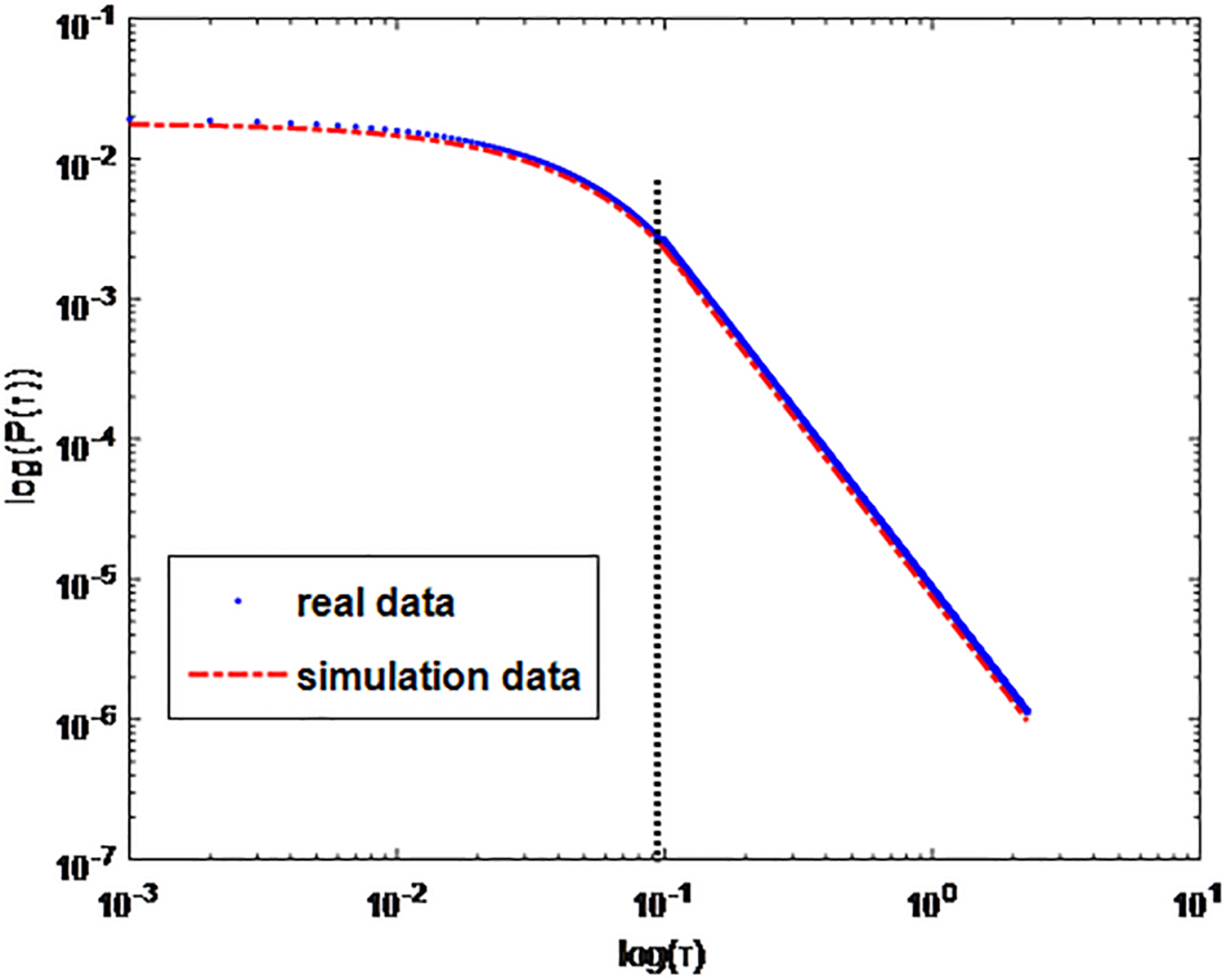
The simulation result compared with the real data. T is set to 0.001s. The result is obtained with parameter values $\frac{1}{\alpha }=1.483,\tau_{0}=0.1s,\lambda =20.77,\beta_{2}=7.5*e^{-06}$.

To verify the accuracy of this combination model, we simulate the actual data. We use the data of Ali-talk in dataset A, and calculate the *P*(*τ*). Ali-talk is an app which is widely used by users of Taobao. According to the *P*(*τ*) obtained by real data, we can get the parameters *α*, *τ*_0_, *β*_2_ and *λ*. Then using Eq (11) and *β*_1_ which we calculated by Eq (10) we get similar results between Alitalk data and simulation data and shown in Fig 19.

**Fig 20:**
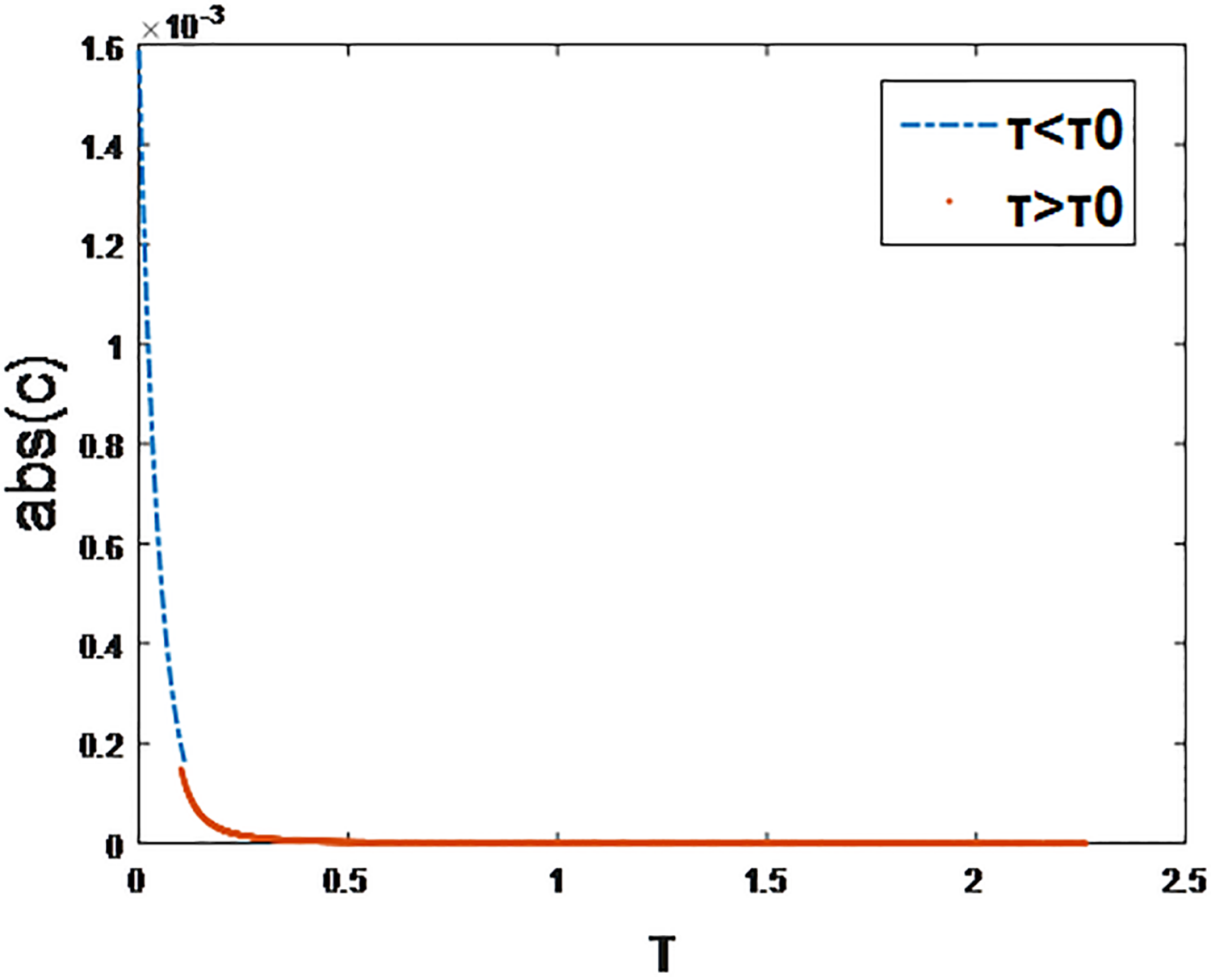
The difference between real data and simulation data. The x label represents the inter-event time *τ* and the y label represents the absolute value of difference between real data and simulation data.

Fig 20 represents the difference between real data and simulation data shown in Fig 3. We can see the difference between Ali-talk data and simulation data is less than 1.6*10^−3^. It verifies that the combination model can catch the heterogeneity of IM services users properly.

## Conclusion

This paper investigates the inter-event time distribution of QQ and WeChat in two cities, and reveals that the inter-event time distributions of QQ and WeChat in both cities follow a piecewise distribution of exponential and power-law distribution when the *T* is set to 0.001s, thus indicating that the online behavior of IM services users’ are heterogeneous in different time scales. The phenomena may be caused by the communication mechanism of IM services and the hobby of users. The simulation results verify that the combination model proposed in the paper can describe the heterogeneity of IM services users properly. The new finding is useful for the application of information diffusion, disease infection, prediction of the economic development of a City, research on the mechanism of IM services, and so on.

Though the inter-event time distribution of QQ in the two cities follows a piecewise distribution, the parameters of the fitting distribution in two cities are distinct. As is referred to in [17], the behavior pattern of IM users is closely correlated with the development of the economy, transportation and communication in the same area. It’s promising to explore the relationship between inter-event time distribution of QQ in one city and the city index. So that we can get the city index easily.

## Supporting information

S1 Supporting Information File A. The experimental data of city A. File B. The experimental data of city B. File C. The experimental data of verification.
(RAR)
